# International classification of functioning, disability and health (ICF) in daily clinical practice: Structure, benefits and limitations

**DOI:** 10.1192/j.eurpsy.2021.198

**Published:** 2021-08-13

**Authors:** C. Krzoska

**Affiliations:** Praxis Dipl.-psych. Carolin Krzoska, Sanamens Praxisgemeinschaft Theil | Krzoska, Helmstedt, Germany

**Keywords:** mental health, ICF, Intellectual Disabilities, Social Medicine

## Abstract

**Introduction:**

The diagnosis of intellectual disability (ID) alone does not predict the level of required care, functional outcomes or limitations in social and occupational participation. The International Classification of Functioning, Disability and Health (ICF) is a taxonomy of health and health-related domains. It provides a common language and framework for describing the level of functioning of a person within their unique environment. Furthermore, it helps to describe health problems of a person in line with the International Classification of Diseases (ICD-10).

**Objective:**

Introducing the ICF taxonomy exemplary in the care of individuals with ID and mental health problems in Germany.

**Method:**

Comparison of the ICF’s comprehensive multidisciplinary approach to assess an individual’s level of functioning and care in relation to assessing the needs of persons with ID based on clinical experience.

**Results:**

The ICF provides a standardised assessment instrument to determine individual functional needs for the care, rehabilitation and societal integration of individuals with disabilities, which is a statutory requirement in many European countries.

**Conclusion:**

Using the ICF for the assessment and management of patients with chronic health conditions, mental disorders and ID can help to accurately define individual therapeutic goals and monitor functional outcomes. A comprehensive narrative description of the patient’s functional status and clinical needs is comparatively time-consuming, requires greater effort by the assessing clinician and carries a higher risk of omission of pertinent functional domains; furthermore, a single ICF item confers little additional benefit to the patient in terms of the treatment or care they subsequently receive.

**Disclosure:**

No significant relationships.

